# Socioeconomic background, exam performance, and probability of medical school application among all MCAT examinees, 2017 to 2019

**DOI:** 10.1186/s12909-026-08940-2

**Published:** 2026-03-29

**Authors:** Arman A. Shahriar, Robert Englander, Yan Che, Keme Carter

**Affiliations:** 1 Present Address: Indian Health Service, 24760 Hospital Rd, Red Lake, MN 56671 USA; 2 Department of Medicine, The University of Chicago Medical Center, Chicago, IL USA; 3 University of Minnesota Medical School, Minneapolis, MN USA; 4 University of Illinois College of Medicine, Chicago, IL USA; 5 Department of Public Health Sciences, University of Chicago, Chicago, IL USA; 6 The University of Chicago Pritzker School of Medicine, Chicago, IL USA

**Keywords:** Medical education, MCAT, Socioeconomic diversity, Medical school application, Admissions

## Abstract

**Background:**

The Medical College Admissions Test (MCAT) remains a significant barrier for students from low socioeconomic status (SES) backgrounds, with performance disparities driven by unequal access to resources rather than by differences in aptitude. Holistic admissions practices seek to contextualize exam performance alongside other essential pre-medical competencies such as resilience, empathy, and scientific inquiry. Evidence shows that students with middle-tercile MCAT scores who demonstrate these qualities will succeed in medical school and beyond. However, only those who apply can benefit from such holistic review. Little is known about application behaviors at this pivotal stage, when examinees must decide whether to apply with their achieved MCAT score. This study examines the relationship between socioeconomic status, MCAT performance, and application probability.

**Methods:**

This study analyzed de-identified Association of American Medical Colleges (AAMC) data for all MCAT examinees between 2017 and 2019, including sociodemographic characteristics and total MCAT score. The outcome was allopathic (M.D.) medical school application status through the 2021 application cycle, allowing examinees at least two full years to apply. The AAMC parental education and occupation (EO) indicator stood as a proxy for SES – ranging from EO-1 (less than college degree, any occupation) to EO-5 (doctoral degree; executive, managerial or professional occupations). Socioeconomic disadvantage (SED) was defined as EO-1 and EO-2.

**Results:**

Middle- or top-tercile MCAT scores were achieved by 51.8% of examinees with SED (middle, 33.2%; top, 18.6%) compared with 73.1% of examines without SED (middle, 34.4%; top, 38.7%). The probability of applying to medical school was closely tied to MCAT performance, and regardless of SES, the probability of application with any given MCAT score was similar. While most top-tercile performers (92 to 94%) applied, a much lower proportion of middle-tercile performers (78 to 80%) applied.

**Conclusions:**

Socioeconomically disadvantaged MCAT examinees infrequently score in the top tercile where application rates are uniformly high. Those with acceptable, middle-tercile performance may under-appreciate their academic achievements through economic adversity, as over 20% do not apply to medical school. Targeted interventions are needed to promote application and maximize the impact of holistic review.

**Supplementary Information:**

The online version contains supplementary material available at 10.1186/s12909-026-08940-2.

## Introduction

Workforce diversity improves the quality of our health care system and remains a top priority within organized medicine [1, 2]. However, prominent sociodemographic diversity gaps persist, in part because the profession remains less accessible to students of low socioeconomic status (SES) who face disproportionate academic, financial, and social barriers along the pipeline [3–7]. One key barrier is the Medical College Admission Test (MCAT), where sociodemographic performance disparities may reflect structural disadvantages – such as differential access to preparatory resources – rather than differences in aptitude [5, 7–9].

Holistic admissions practices aim to account for applicant structural disadvantages [10], but their impact is limited to students who apply. The selection of candidates who will succeed in medical school and go on to become high caliber physicians is multifactorial and includes assessments of academic readiness (e.g., MCAT score) [11] alongside other competencies such as scientific inquiry, empathy, communication skills, resilience, and cultural humility [12–14]. Indeed, students with middle-tercile MCAT scores who demonstrate these other qualities succeed at high rates in medical school – graduating on time and passing licensing exams on the first attempt [14]. Despite this, MCAT examinees rank academic qualifications alongside costs as the top factor discouraging application [15].

The Association of American Medical Colleges (AAMC) routinely reports aggregate academic credentials (e.g., grades and MCAT scores) for both applicants and matriculants [16], but little is known about the academic credentials and sociodemographic characteristics of examinees who forgo application despite their demonstrated interest in medicine by virtue of reaching this stage. To address this gap in knowledge at a critical juncture and potentially inform up-stream interventions that minimize the loss of diverse and qualified applicants, this study examined the relationship between MCAT performance and application according to SES.

## Method

### Study data

This cross-sectional study utilized licensed deidentified data from the Association of American Medical College (AAMC) and American Medical College Application Service® (AMCAS) server, as well as the MCAT data team, and was deemed exempt by the University of Chicago Institutional Review Board. We followed the Strengthening the Reporting of Observational Studies in Epidemiology (STROBE) guidelines for observational research and provide the American Association for Public Opinion Research (AAPOR) Standard Disclosure Checklist for Survey Research (full Table provided in the Supplemental Digital Appendix).

For all unique MCAT examinees between 2017 and 2019, we extracted total MCAT score (using the highest score if multiple attempts made), application status (through the 2021 cycle), US citizenship status, sex, self-identified race-ethnicity, and the parental education-occupation (EO) category – a validated surrogate for socioeconomic status (SES) available for most examinees who are US citizens or permanent residents [17, 18]. Examinees are classified into mutually exclusive categories EO-1 through EO-5 based on their responses to survey items concerning highest parental level of education and parental occupation, from EO-1 = less than college degree (any occupation) to EO-5 = doctoral degree; executive, managerial or professional occupations. We excluded those who were not US citizens or permanent residents from our analysis due to differing experiences and significant unavailability of covariates such as the EO-category. We also excluded examinees without a scored MCAT.

Aggregate sociodemographic groups were generated for analysis to reflect structural disadvantage with respect to socioeconomic status or race-ethnicity. Socioeconomic disadvantage (SED) was defined as EO-1 and EO-2 [18]. Structural disadvantage reflective of a marginalized racial or ethnic identity was defined as racial and ethnic groups currently underrepresented in medicine (URiM). The URiM group included those identifying as Black (non-Hispanic), American Indian (AI) or Alaska Native (AN, non-Hispanic), Native Hawaiian (NH) or Pacific Islander (PI, non-Hispanic), or Hispanic ethnicity (any race). The non-URiM group included White (non-Hispanic) and Asian (non-Hispanic).

### Data analysis

For the entire sample and various sociodemographic groups, we calculated MCAT score distributions (mean, standard deviation), as well as the proportion of examinees who achieved MCAT scores within discrete 2-score bins, and higher-level pertinent MCAT terciles (bottom, middle, and top). Scores exactly at each tercile cutoff were included in the lower tercile bin.

First, to visualize crude application patterns according to SES, we computed the proportion of individuals applying for medical school at each unique MCAT score and fitted smooth loess curves for each EO group [19] – a nonparametric statistical method that has the advantage of not making any assumptions about the relationship between the MCAT scores and the likelihood of an application to medical school. Then, to contextualize findings, we generated MCAT score histograms and used stacked bar plots to depict the proportion who applied within each MCAT bin.

To make statistical comparisons in application rates across subgroups of interest, we first examined unadjusted probabilities of application across each covariate (MCAT score tercile [bottom, middle, top], exam year, sex, EO-category [individual groups], and race-ethnicity [individual groups]) and made comparisons using *X*^*2*^tests with statistical significance set at p-value < 0.05. Then, multivariable logistic regression models were applied, adjusting for all other covariates, including MCAT year, sex, URiM status, or SED status. Adjusted odds ratios (aOR) with 95% confidence intervals are reported alongside application probabilities with 95% confidence bands. Despite the outcome being common, we chose to present aOR (instead of adjusted relative risks) to include more adjustment variables and avoid the introduction of additional assumptions that may not be valid. We focused the adjusted analysis around examinees with an overall middle-tercile performance given the greater variability in application rates in this group, and compelling evidence these students succeed in medical school and improve diversity [14]. Reference groups were set as those with middle-tercile performance and structural disadvantage (URiM or SED) who we anticipated would apply at higher rates than counterparts who are not structurally disadvantaged with comparable performance. All statistical analyses were performed from June 5, 2024, to April 10, 2025, using R and RStudio (Version 2024.04.2, R Foundation for Statistical Computing, Vienna, Austria) and the Tidyverse packages [20].

## Results

### Sample demographics

Between 2017 and 2019, there were 188,303 unique MCAT examinees. After excluding 25,935 (13.8%) who were not US citizens or permanent residents and an additional 519 (0.3%) for whom exam scores were unavailable, the final sample consisted of 161,849 unique examinees (Table 1): 54.7% female, 22.4% URiM (Black, 9.0%; Hispanic, 13.1%; AI/AN, 0.23%; NH/PI, 0.10%), 77.6% non-URiM (White, 48.2%, Asian, 22.4%, Other 7.0%), 31.7% with socioeconomic disadvantage (EO-1, 23.0%; EO-2, 8.7%), 68.3% without socioeconomic disadvantage (EO-3, 21.9%; EO-4, 20.6%; EO-5, 25.8%).

**Table 1:** Sample demographics and crude probabilities of application

Variable	N (%)	% Variable^a^	Applied (95% CI)	*p*-value^b^
All	161,849 (100.0)		0.714 (0.711—0.717)	
MCAT Score Tercile				< 0.001
Top	50,514 (31.3)		0.936 (0.933—0.938)	
Middle	54,181 (33.6)		0.789 (0.786—0.793)	
Bottom	57,154 (35.4)		0.418 (0.414—0.422)	
Exam year				< 0.001
2017	52,142 (32.2)		0.733 (0.729—0.736)	
2018	54,212 (33.5)		0.719 (0.715—0.722)	
2019	55,495 (34.3)		0.691 (0.687—0.695)	
Sex				< 0.001
Female	88,232 (54.5)	54.7%	0.702 (0.699—0.705)	
Male	73,122 (45.2)	45.3%	0.728 (0.725—0.731)	
Other/Missing	495 (0.3)		0.671 (0.628—0.711)	
EO Category				< 0.001
SED	47,041 (29.1)	31.7%		
EO-1	34,148 (21.1)	23.0%	0.625 (0.620—0.630)	
EO-2	12,893 (8.0)	8.7%	0.678 (0.670—0.686)	
No SED	101,206 (62.5)	68.3%		
EO-3	32,386 (20.0)	21.9%	0.713 (0.708—0.718)	
EO-4	30,566 (18.9)	20.6%	0.756 (0.799—0.807)	
EO-5	38,254 (23.6)	25.8%	0.803 (0.799—0.807)	
Missing	13,602 (8.4)		0.621 (0.665—0.681)	
Race and ethnicity				< 0.001
URiM	35,318 (21.8)	22.4%		
AI/AN, non-Hispanic	369 (0.23)	0.23%	0.602 (0.561—0.712)	
Black, non-Hispanic	14,180 (8.8)	9.0%	0.673 (0.665—0.681)	
Hispanic	20,620 (12.7)	13.1%	0.636 (0.630—0.643)	
NH/PI, non-Hispanic	149 (0.09)	0.09%	0.640 (0.561—0.712)	
Non-URiM	122,044 (75.4)	77.6%		
Asian, non-Hispanic	35,193 (21.7)	22.4%	0.729 (0.724—0.734)	
Other, non-Hispanic	10,918 (6.7)	6.9%	0.738 (0.730—0.746)	
White, non-Hispanic	75,933 (46.9)	48.3%	0.731 (0.728—0.734)	
Missing	4,487 (2.8)		0.732 (0.719—0.745)	

*Abbreviations*: AI/AN – American Indian/Alaskan Native, *NH/PI* Native Hawaiian/Pacific Islander, *EO* parental education and occupation indicator, *URiM* Under-represented in medicine, *SED* Socioeconomic disadvantage

^a^Percentages of key variables after missing values removed

^b^X^2^ test of comparison with significance set at *p* < 0.05

### MCAT score distributions

**Fig. 1:**
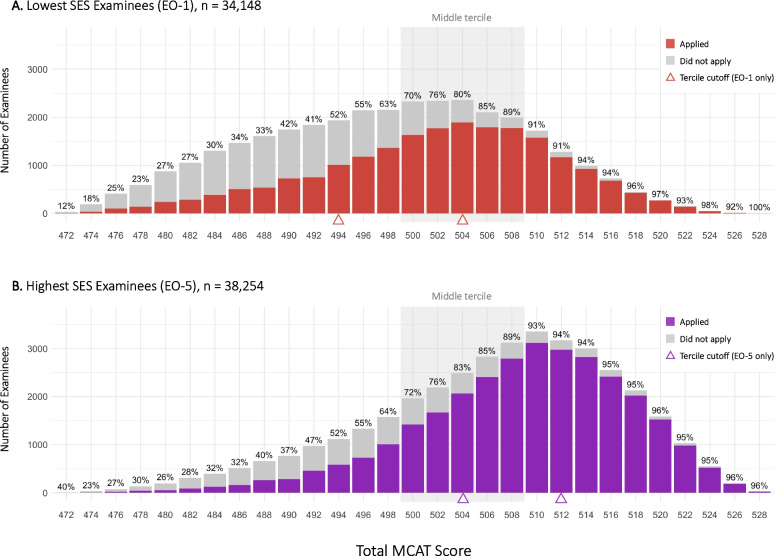
Probability of Application and Underlying Score Distributions at Extremes of SES (EO-1 and EO-5), *N =* 72,402. Authors’ analysis of data from the Association of American Medical Colleges (AAMC) and American Medical College Application Server (AMCAS). Percentages above each bar denote the proportion of examinees within the bin range who applied, as represented by the dark shade. Gray shade indicates the middle tercile of scores overall (499 to 509). Triangles denote tercile cutoffs within each specific EO category (e.g., the cutoff for a top tercile score among only EO-1 examinees was 504, whereas the cutoff for a top-tercile score among only EO-5 examinees was 512). The EO categories are derived from AAMC and AMCAS survey items concerning parental education (E) and occupation (O). Categories range from EO-1 = less than college degree (any occupation) to EO-5 = doctoral degree; executive, managerial or professional occupations

Total MCAT scores ranged from 472 to 528 with a median of 504, a mean of 503.0 and standard deviation (SD) of 11.0. Score distributions were similar across exam years but varied significantly across sociodemographic groups. According to sex, the mean (SD) was 501.5 (11.1) for female, and 504.6 (10.7) for male. According to race-ethnicity: 496.8 (9.7) for AI/AN, 505.3 (11.0) for Asian, 494.9 (10.5) for Black, 497.7 (11.2) for Hispanic, 504.7 (9.9) for White, 498.0 (10.6) for NH/PI, and 503.4 (10.7) for all other. Finally, according to SES: EO-1, 498.6 (10.6); EO-2, 501.1 (10.8); EO-3, 503.2 (10.4); EO-4, 505.3 (10.3); and EO-5, 507.2 (9.9). Score distributions at the two extremes of SES (EO-1 and EO-5), which together comprise 48.9% of the sample, are depicted in Fig. 1. Score distributions for the remaining three SES groups (EO-2, EO-3, and EO-4) are included in the Supplemental Digital Appendix.

### MCAT score terciles

**Fig. 2:**
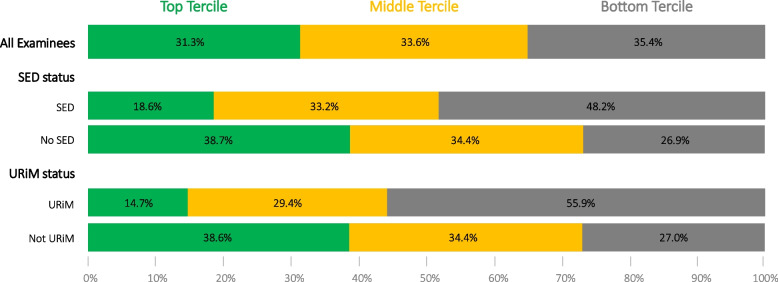
MCAT Performance (Terciles) by Indicators of Structural Disadvantage: Socioeconomic Disadvantage (SED) Status and Underrepresented in Medicine (URiM) Status. Authors’ analysis of data from the Association of American Medical Colleges (AAMC) and American Medical College Application Server (AMCAS). Bars denote the proportion of examinees within each tercile. The top, middle, and bottom terciles ranged from 510–528, 499–509, and 472–498, respectively. Examinees at tercile cutoffs were grouped in the lesser of the two terciles. Socioeconomic disadvantage (SED) was defined using parental education and occupation (EO) categories derived from self-reported parental education and occupation, with EO-1 and EO-2 considered SED. Race and ethnicity were self-reported by examinees and categorized into mutually exclusive groups. Underrepresented in medicine (URiM) defined as Hispanic or Black (non-Hispanic), American Indian/Alaska Native (non-Hispanic), or Native Hawaiian/Pacific Islander (non-Hispanic). Non-URiM defined as White (non-Hispanic) or Asian (non-Hispanic)

Middle- or top-tercile MCAT scores were achieved by 51.8% of examinees with SED (middle, 33.2%; top, 18.6%), 73.1% of those without SED (middle, 34.4%; top, 38.7%), 44.2% of URiM (middle, 29.4%; top, 14.7%), and 73.0% of non-URiM (middle, 34.4%; top, 38.6%), p < 0.001 (Fig. 2). Disaggregated tercile analysis according to individual racial and ethnic groups is available in the Supplemental Digital Appendix.

### Crude application rates

**Fig. 3:**
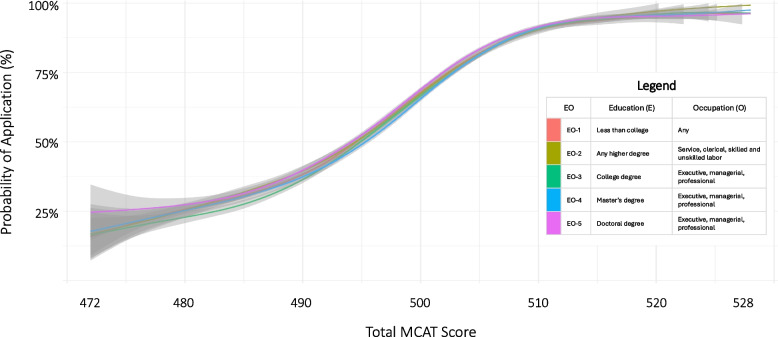
Probability of Medical School Application Through 2022 According to MCAT Score and Socioeconomic Status (EO category), Examinees 2017 to 2019. Authors’ analysis of data from the Association of American Medical Colleges (AAMC) and American Medical College Application Server (AMCAS). The EO categories are derived from AAMC and AMCAS survey items concerning parental education (E) and occupation (O). Category definitions are included in the figure legend. Curves represent locally weighed regression (LOESS) along with 95% confidence bands to summarize the trends. Curves display associations between highest total MCAT score and the probability of applying for medical school through the 2021 cycle

The overall application rate through the 2021 application cycle was 71.4% with significant variability by MCAT tercile (bottom, 41.8%; middle, 78.9%; top, 93.6%), SES, race-ethnicity, sex, and exam year (Table 1). There was a clear dose–response relationship between MCAT score and probability of application with little variability by SES (Fig. 3). At the extremes of SES (EO-1 and EO-5), alongside prominent differences in score distributions (Fig. 1), there were large differences in proportion who applied (EO-1, 21,448/34,316 (62.5%) vs EO-5, 30,814/38,358 (80.3%), p < 0.001).

### Multivariate analysis: adjusted odds of application

Among examinees with middle-tercile MCAT performance, those with SED (EO-1 and EO-2) applied at slightly lower rates than those without SED (78.2% vs 79.3%; aOR 0.91 [0.87–0.95], Table 2).

**Table 2:** Probability and adjusted odds of medical school application by aggregate sociodemographic group and total MCAT score tercile, 2017 to 2019

Sociodemographic group	MCAT Tercile^a^	N (%)	App. Probability (95% CI)^b^	aOR (95% CI)^c^
All examinees	All	161,849 (100.0)	0.714 (0.711—0.717)	
	Bottom	57,154 (35.4)	0.418 (0.414—0.422)	
	Middle	54,181 (33.6)	0.789 (0.786—0.793)	
	Top	50,514 (31.3)	0.936 (0.933—0.938)	
Socioeconomic status – SED status				
No SED (EO 3–5)	All	101,206 (100.0)	0.761 (0.758—0.764)	
	Bottom	27,251 (26.9)	0.428 (0.422—0.434)	0.179 (0.173—0.186)
	Middle	34,779 (34.4)	0.793 (0.789—0.797)	reference
	Top	39,176 (38.7)	0.938 (0.936—0.940)	4.159 (3.965—4.364)
SED (EO 1–2)	All	47,041 (100.0)	0.641 (0.638—0.644)	
	Bottom	22,695 (48.2)	0.409 (0.402—0.416)	0.156 (0.150—0.162)
	Middle	15,575 (33.2)	0.782 (0.775—0.788)	0.905 (0.865—0.948)
	Top	8,771 (18.6)	0.926 (0.921- 0.931)	3.379 (3.119—3.667)
Race-ethnicity – URiM status				
Non-URiM	All	122,044 (100.0)	0.680 (0.677—0.683)	
	Bottom	32,983 (27.0)	0.384 (0.379—0.389)	0.180 (0.174—0.187)
	Middle	42,016 (34.4)	0.776 (0.772—0.780)	reference
	Top	47,045 (38.6)	0.936 (0.934—0.938)	4.152 (3.970—4.344)
URiM^d^	All	35,318 (100.0)	0.624 (0.621—0.627)	
	Bottom	19,714 (55.8)	0.474 (0.467—0.480)	0.271 (0.261—0.282)
	Middle	10,398 (29.4)	0.847 (0.840—0.853)	1.625 (1.529—1.729)
	Top	5,206 (14.7)	0.936 (0.929—0.942)	4.355 (3.877—4.909)

Reference groups: For SES, the reference group was chosen as examinees with middle-tercile MCAT scores without SED (EO-3 through EO-5). For race-ethnicity, the reference group was examinees with middle terciles MCAT scores who identified as non-URiM

^a^Terciles include those excluded from analysis (non-US citizens or permanent residents)

^b^Probability of application through the American Medical College Application Service (AMCAS) server through the 2021 application cycle

^c^Multivariate model including exam year, sex, aggregate race-ethnicity (URiM and non-URiM), aggregate socioeconomic status (SED and no SED)

^d^Aggregate groups (URiM and non-URiM). Race and ethnicity were self-reported and categorized in mutually exclusive groups as American Indian or Alaska Native (AI/AN), Asian (non-Hispanic), Black (non-Hispanic), Hispanic, Native Hawaiian or Pacific Islander (NH/PI), White (non-Hispanic), or Other (as selected by the individual). Race and ethnicity were further categorized as URiM (Black, Hispanic, AI/AN, NH/PI) or non-URiM (White, Asian)

## Discussion

There is a growing collective awareness surrounding the importance of SES representation in medicine [3–5], but the profession remains less accessible to low-SES students who face myriad financial, economic, and social obstacles along the educational continuum. In this cross-sectional study of all MCAT examinees from 2017 to 2019, the probability of an ultimate medical school application was closely tied to MCAT performance. Across the axis of SES, while there were prominent differences in MCAT performance, the probability of applying with any given MCAT score remained similar. Top tercile scores – where application rates are uniformly high – are relatively uncommon for low-SES examinees and may reflect structural inequities, including differential access and utilization of preparatory resources, weaker academic support systems, and other non-academic stressors or responsibilities throughout life and during the exam preparation period [5, 7–9, 17, 21]. Although middle-tercile scores – which low-SES examinees more commonly achieved – also forecast success [14], we found that over one-fifth (21.8%) of low-SES examinees (EO-1 and EO-2) with middle-tercile scores did not apply.

These findings suggest that low-SES pre-medical students who have navigated structural adversity to reach this final stage of the pipeline, and performed adequately on the MCAT, may undervalue their achievements. Indeed, URiM students also encounter structural barriers [8] – some of which stem from the tight relationship between SES and race [22] – and infrequently score in the top tercile, but the higher application rates among URiM with middle-tercile performance suggest a heightened awareness of the barriers they have overcome. Although cost is also cited by MCAT examinees as an application deterrent [15], the consistency of the relationship between MCAT score and application probability across SES groups suggests that an individuals’ perceived academic competitiveness may play a more influential role in the decision to apply.

This study identifies application submission as a novel, upstream barrier to improving sociodemographic representation in medicine. Both systemic and individual-level interventions are needed to improve application rates among low-SES students with acceptable (middle-tercile) MCAT performance – targeting the application rates of top-tercile performers (above 90%). Such attention to SES is particularly relevant given the recent overturning of race-conscious admissions by the Supreme Court [3, 23] and caps on federal borrowing for graduate students [24].

At the system level, the AAMC should stratify its annual reports [16] of applicant and matriculant academic credentials by SES indicators (e.g., EO category, fee assistance) and may even consider distributing these reports electronically to all examinees alongside score reports. To guide individual-level efforts, the AAMC could provide undergraduate institutions with lists of examinees who meet criteria for socioeconomic disadvantage. At the individual level, premedical advisors should proactively discuss socioeconomic background with advisees. Advisors can provide both psychological support (e.g., contextualizing academic performance relative to similar-SES peers) and financial support (e.g., strategies to mitigate cost-related barriers) to encourage application [5]. Doing so could introduce thousands of resilient, underrepresented [4, 5] candidates into the annual applicant pool, thereby maximizing the impact of existing admissions initiatives (e.g. holistic review) [10] designed to evaluate applicants’ lived experiences and other key attributes alongside traditional academic metrics.

This study had several limitations. First, we were unable to ascertain osteopathic (D.O.) application status from the AAMC and AMCAS databases, limiting our ability to characterize the true applicant pool. Second, the limited number of available covariates raise the possibility of confounding – the most significant being undergraduate GPA data which are not available through the AAMC and AMCAS for non-applicants. Third, our study treated application as a binary variable, but there may also be socioeconomic disparities in the number of applications submitted by any given applicant which may impact the probability of acceptance and matriculation [25]. Finally, the EO indicator, while a validated SES proxy, has limitations [17]. Future research leveraging longitudinal AAMC survey data, particularly the post-MCAT questionnaire, may further elucidate the factors influencing application decisions among low-SES examinees.

## Supplementary Information

Supplementary Material 1.

## Data Availability

Data for this analysis were licensed by the University of Minnesota and University of Chicago from the AAMC. Data are proprietary and cannot be made available to outside investigators without entering a data licensing agreement (DLA). The authors and the AAMC will consider such an agreement on a case-by-case basis.
