# Supplementary material for: Socioeconomic background, exam performance, and probability of medical school application among all MCAT examinees, 2017 to 2019
Source: BMC Med Educ. 2026 Mar 29;26:727. doi: 10.1186/s12909-026-08940-2 (PMC13151120; doi:10.1186/s12909-026-08940-2)

**Online Only Supplementary Appendix**

**Methods**

**Table.** AAPOR Standard Disclosure Checklist for Survey Research

**Results**

**Table.** Race-ethnicity and Application Probability by MCAT Tercile

**Figure.** Score Distribution and Application Histograms for EO-2, EO-3, and EO-4

**Table 1. AAPOR Standard Disclosure Checklist for Survey Research**

| **Basic Disclosure Element** | **Details** |
| --- | --- |
| Survey sponsor | Association of American Medical Colleges (AAMC) |
| Survey/Data collection supplier | AAMC and American Medical College Application Service® (AMCAS), as well as MCAT data team |
| Population represented | All unique MCAT examinees from 2017 to 2019 who were US citizens or permanent residents with scored MCAT exams |
| Sample size | All unique examinees 2017-2019: 188,303  Study sample: 161,849 (86.0%) |
| Mode of data collection | Electronic survey  Scored MCAT exams |
| Start and end dates of data collection | January 1, 2017 – December 31, 2021 |
| Margin of sampling error for total sample | NA |
| Margin of sampling error for key subgroups | NA |
| Are the data weighted? | NA |
| Contact for more information | **Tom Massari, MPS**  Senior Research and Data Analyst, AAMC  tmassari@aamc.org |

**Table. Race-ethnicity and Application Probability by MCAT Tercile**

| **Race-ethnicity** | **MCAT Tercile** | **N (%) ^a^** | **Applied (95% CI)** |
| --- | --- | --- | --- |
| AI/AN | Bottom | 221 (59.9) | 0.48 (0.415 - 0.545) |
| AI/AN | Middle | 109 (29.5) | 0.743 (0.654 - 0.816) |
| AI/AN | Top | 39 (10.6) | 0.897 (0.764 - 0.959) |
| Asian | Bottom | 9287 (26.4) | 0.362 (0.352 - 0.372) |
| Asian | Middle | 10778 (30.6) | 0.762 (0.754 - 0.77) |
| Asian | Top | 15128 (43.0) | 0.934 (0.93 - 0.937) |
| Black | Bottom | 8822 (62.2) | 0.528 (0.517 - 0.538) |
| Black | Middle | 3951 (27.9) | 0.897 (0.887 - 0.906) |
| Black | Top | 1407 (9.9) | 0.957 (0.945 - 0.967) |
| Hispanic | Bottom | 10593 (51.4) | 0.428 (0.419 - 0.438) |
| Hispanic | Middle | 6290 (30.5) | 0.817 (0.808 - 0.827) |
| Hispanic | Top | 3737 (18.1) | 0.928 (0.919 - 0.936) |
| NH/PI | Bottom | 78 (52.3) | 0.487 (0.379 - 0.596) |
| NH/PI | Middle | 48 (32.2) | 0.729 (0.59 - 0.834) |
| NH/PI | Top | 23 (15.4) | 1 (0.857 - 1) |
| White | Bottom | 20308 (26.7) | 0.384 (0.377 - 0.391) |
| White | Middle | 27569 (36.3) | 0.778 (0.774 - 0.783) |
| White | Top | 28056 (36.9) | 0.937 (0.934 - 0.939) |
| All other | Bottom | 3388 (31.0) | 0.447 (0.431 - 0.464) |
| All other | Middle | 3669 (33.6) | 0.798 (0.784 - 0.810) |
| All other | Top | 3861 (35.4) | 0.938 (0.93 - 0.945) |
| Missing | Bottom | 1253 (27.9) | 0.435 (0.408 - 0.463) |
| Missing | Middle | 1536 (34.2) | 0.76 (0.738 - 0.78) |
| Missing | Top | 1698 (37.8) | 0.928 (0.915 - 0.939) |
| Total |  | 161849 |  |

^a^ Percentages indicate proportion of racial-ethnic group within each tercile

**Figure. Score Distribution and Application Histograms**

**Panel A.** EO-2 Examinees, N = 12,893

**
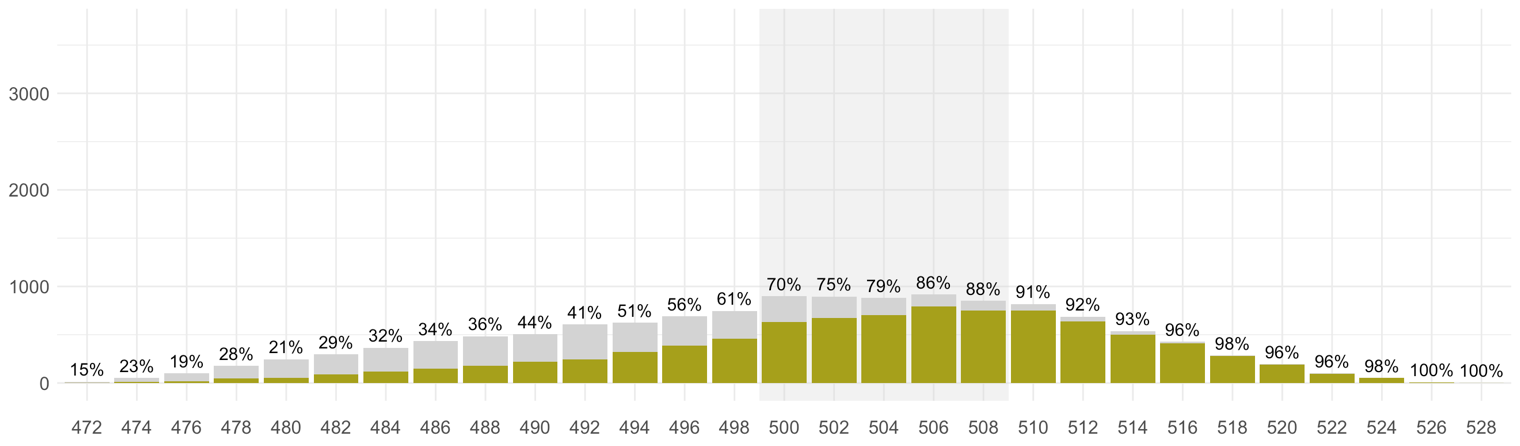
**

**Panel B.** EO-3 Examinees, N = 32,386

**
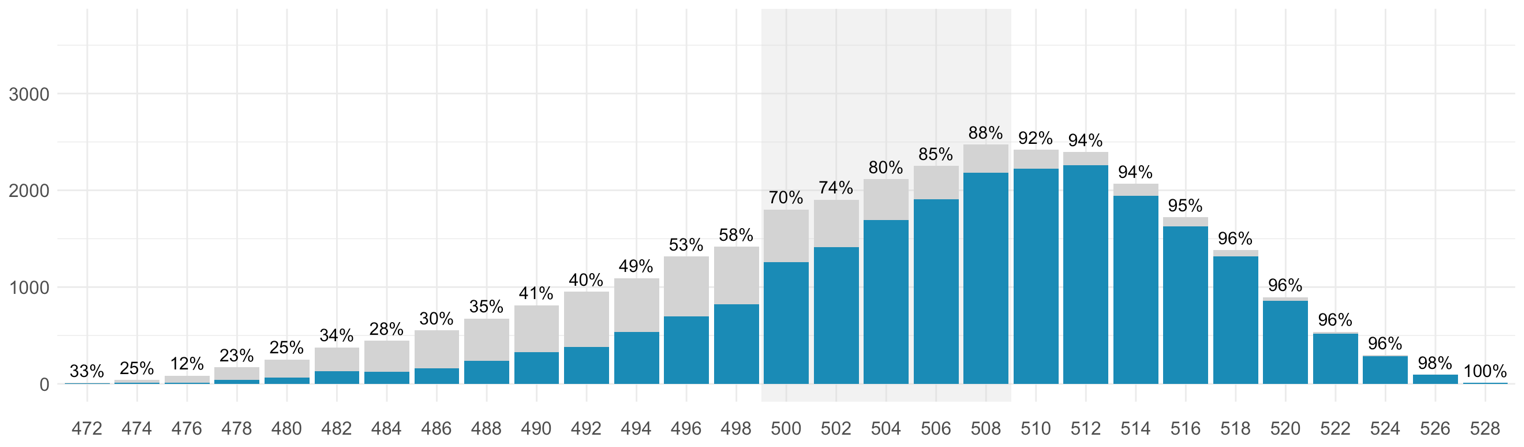
**

**Panel C.** EO-4 Examinees, N = 30,566


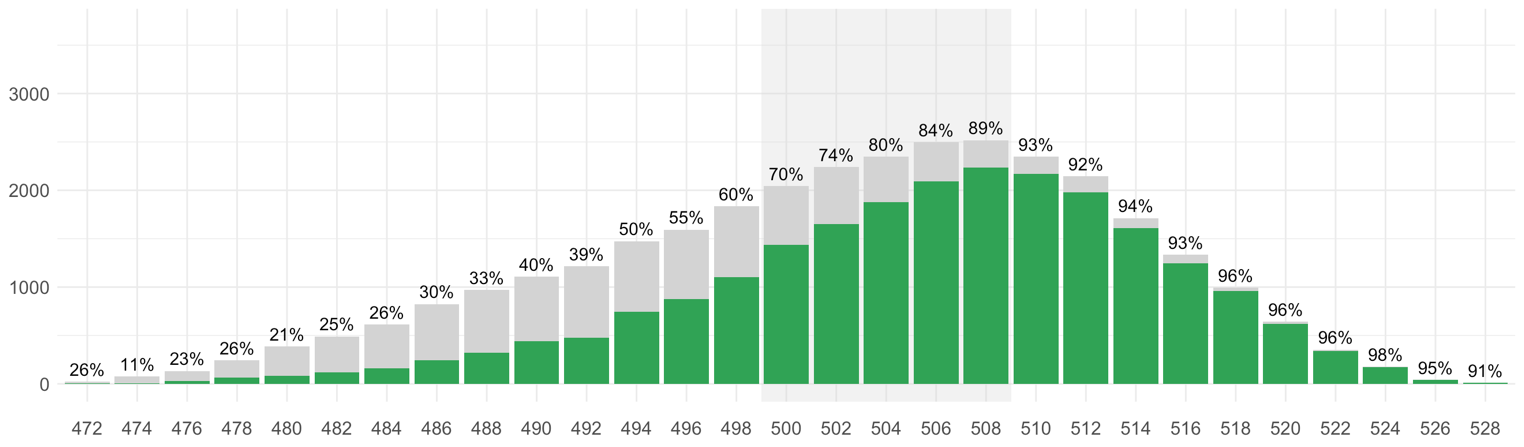

Supplement: Supplementary file 1 — Supplementary Material 1. [file 12909_2026_8940_MOESM1_ESM.docx]
